# Erratum

**DOI:** 10.1590/0037-8682-2012000300023

**Published:** 2021-04-12

**Revista da Sociedade Brasileira de Medicina Tropical/*Journal of the Brazilian Society of Tropical Medicine***

**Title:** Natural infection of triatomines (Hemiptera: Reduviidae) by trypanosomatids in two different environments in the municipality of Ouro Preto do Oeste, State of Rondônia, Brazil

**Vol. 45(3): 2012 - Page: 395-398, - doi:** 10.1590/S0037-86822012000300023

In regard to the triatomines collected in the present study, where we cite: 

*Rhodnius prolixus* and *R. prolixus*.

**Should read:**

*Rhodnius montenegrensis* and *R. montenegrensis*.

